# Mechanical and Surface Properties of Edible Coatings Elaborated with Nanoliposomes Encapsulating Grape Seed Tannins and Polysaccharides

**DOI:** 10.3390/polym15183774

**Published:** 2023-09-15

**Authors:** Angela Monasterio, Emerson Núñez, Natalia Brossard, Ricardo Vega, Fernando A. Osorio

**Affiliations:** 1 Department of Food Science and Technology, Technological Faculty, University of Santiago—Chile, USACH. Av. El Belloto 3735, Estación Central, Santiago 9170022, Chile; angela.monasterio@usach.cl; 2 Department of Fruit Production and Enology, School of Agricultural and Forest Science, Pontificia Universidad Católica de Chile, Av. Vicuña Mackenna 4860, Macul, Santiago 7820436, Chile; ennunez@uc.cl (E.N.); ndbrossa@uc.cl (N.B.); 3 Department of Chemical Engineering, Engineering Faculty, University of Santiago—Chile, USACH. Av. L.B. O’Higgins 3363, Estación Central, Santiago 9170022, Chile; ricardo.vega@usach.cl

**Keywords:** edible coatings, biopolymers, nanoliposomes, surface, polysaccharides, texture

## Abstract

Edible composite coatings (ECC) formulated from biopolymers that incorporate antioxidant molecules represent an innovative alternative to improve food texture and provide health benefits. Tannins have aroused great interest due to their ability to stabilize suspensions and counteract the effects of free radicals. The mechanical and surface properties are crucial to establishing its quality and applicability. In this study, the objective was to analyze the mechanical and surface properties of ECC made with nanoliposomes that encapsulate grape seed tannins (TLS) and polysaccharides such as hydroxypropylmethylcellulose (HPMC) and kappa carrageenan (KCG) for their future direct application in foods susceptible to oxidation. The inclusion of HPMC or KCG affected the density, showing values in the range of 1010 to 1050 [kg/m^3^], evidencing significant changes (*p* < 0.05) in the surface tension in the TLS/FS-HPMC and TLS/FS mixtures. KCG and in the dispersion coefficients, with values in the range of −2.9 to −17.6 [mN/m] in HPS (S_1_) and −17.6 to −40.9 [mN/m] in PDMS (S_2_). The TLS/FS-HPMC coating showed higher stiffness and elastic recovery capacity than the TLS/FS-KCG coating, suggesting that the presence of TLS influenced the stiffness of the polymer. HPMC is recommended as a suitable polymer for coating solids, while KCG is more appropriate for suspensions. These findings provide valuable information for directly applying these ECC compounds to food products, potentially offering better preservation and health benefits.

## 1. Introduction

Edible coatings (EC), thin films applied around foods to protect them from adverse environmental conditions such as light, mechanical damage, and vapors or gases [1], have gained significant attention in current food industry research. The growing demand for foods with enhanced attributes in terms of shelf life, sensory quality, and nutritional properties has driven a continuous quest for innovative solutions to meet consumer demands [2,3,4]. One promising approach is the implementation of EC, which extends food preservation and offers specific functionalities such as texture enhancers, masking agents for unwanted flavors, and carriers for bioactive compounds [5,6]. Ongoing studies on polysaccharide biopolymers, such as HPMC, which form odorless, colorless, and tasteless EC, are focused on developing flexible edible films based on HPMC and antioxidants for application in food packaging materials [7,8,9]. Another polysaccharide that has attracted scientific attention is kappa carrageenan (KCG), which exhibits good mechanical properties, particularly the formation of thermoreversible gels [10,11,12]. These polysaccharides create EC with improved structural and substance transport properties [13].

Tannins are natural sources of antioxidants and can enhance food texture owing to their high fiber content [14,15,16]. Nevertheless, they are susceptible to abrupt changes in temperature and pH, both of which are critical factors in food processing [17]. Furthermore, their interaction with salivary proteins has been reported to lead to astringency and a bitter taste, complicating their direct incorporation into foods [18,19,20]. To address these challenges, the utilization of nanoencapsulation systems employing liposomes, spherical vesicles consisting of a phospholipid bilayer in aqueous solvents, has been proposed to solve them. Liposomes facilitate the retention of antioxidants within their structure thanks to their advantageous carrier properties arising from size and biocompatibility [21]. Incorporating plasticizers, cross-linking agents, emulsifiers, or lipids with biopolymers and antioxidant molecules makes it possible to modify their physicochemical, mechanical, and functional attributes, forming composite edible coatings (ECC). These ECCs are intentionally designed to amplify conventional coatings, affording them structural, mechanical, surface, and transport properties aligned with those of the food matrix [22].

In this context, the objective of this research was to analyze the mechanical and surface properties of ECC elaborated with nanoliposomes encapsulating grape seed tannins (TLS) and polysaccharides, such as HPMC and KCG, intended for direct application in susceptible-to-oxidation foods. These coatings are for direct use on foods, distinct from the studies mentioned above that primarily seek alternatives to replace conventional materials such as plastics.

This study focuses on the interaction between nanoliposomes encapsulating bioactive compounds and polysaccharides in ECC formation for food applications. An in-depth analysis of these coatings’ mechanical and surface properties will provide valuable insights for optimizing their performance and subsequent direct application in food products, thereby contributing to the development of high-quality, extended shelf-life foods. Furthermore, this alternative presents the added advantage of incorporating antioxidant molecules, contributing to potential health benefits.

## 2. Materials and Methods

### 2.1. Materials

Condensed tannin powder with a medium degree of polymerization (mDP) of 2.5 ± 0.2, a galloylation degree (%G) of 15.5 ± 1.1, and an average molecular weight (MW) of 784 ± 61 was obtained from Enartis Sepsa S.A.U. (La Rioja, Spain). Soy lecithin was provided by Dimerco Comercial Ltda. (Santiago, Chile). Glycerol PA (Gly) (purity ≥ 99%) was obtained from Sigma Aldrich (St. Louis, MO, USA). 2,2’-Azino-bis-3-ethylbenzothiazoline-6-sulfonic acid diammonium salt ≥98% (ABTS) from Sigma Aldrich (St. Louis, MO, USA) was used. Ethanol PA (≥99.9%) from Merck KGaA (Darmstadt, Germany) and Milli-Q water were used as solvents. KCG (Carragel PGU 5289, Gelymar^®^, Santiago, Chile) and HPMC (Methocel E19, Dow Wolff Cellulosics, Bomlitz, Germany) were used.

### 2.2. Methods

#### 2.2.1. Antioxidant Suspension (AS)

The antioxidant suspension used was the TLS elaborated using the heating/homogenization method with lamellarity and size reduction by applying cycles of ultrasound [23]. To prepare 100 [mL] of TLS [1 mg/mL], 0.1 [g] of tannins was mixed with 20 [mL] of citrate buffer (0.1 M at pH 3). Subsequently, 1 [g] of phosphatidylcholine (PC) was added, and the mixture was magnetically stirred at 700 [rpm] for 5 [min]. The liposomal suspension was heated in a thermostatically controlled bath at 80 °C for 1 [h]. After this period, 0.76 [g] of glycerol was added as a lipoprotector [24], dissolved in 40 [mL] of buffer, and the mixture was heated again at 80 °C for 1 [h]. At the end of this second heating period, the remaining 40 [mL] of buffer was added to complete the 100 [mL] volume, followed by five vortexing cycles and ten ultrasonication cycles. Ultrasonication was performed using an ultrasonic cell disruptor (HIELSCHER UP100H, Teltow, Germany) equipped with an MS7 Microtip 7 sonotrode (7 mm in diameter, 120 [mm] in length, 130 [W/cm²] acoustic power density) working at 50% amplitude and a maximum of 100 W. To characterize the physicochemical properties of AS, dynamic light scattering (DLS) was applied to measure the mean particle size (MPS) [25], polydispersity index (PDI) [26], and zeta potential (ξ) [27]. The encapsulation efficiency of AS was determined using ultra-high-performance liquid chromatography (UHPLC) by quantifying the catechin and epicatechin monomers [28]. The total phenolic content (TPC) was determined using the Folin-Ciocalteu method [29], the antioxidant capacity was determined using the ABTS radical scavenging assay [30], and the oxygen radical absorbance capacity (ORAC) was determined using the ITM Prt-104 method [31].

#### 2.2.2. Filmogenic Suspension (FS)

A multilevel factorial design—to determine the optimal concentrations of each polysaccharide (HPMC and KCG) for the formation of an FS—was performed with eight experimental runs per polysaccharide; executed in a single block. The factors, levels, and response variables evaluated are shown in Table 1.

In this experimental design, the concentrations of each polysaccharide were varied at three levels. The response variables evaluated were density and surface tension. The design matrix was generated using the software STATGRAPHICS Centurion XVI, v.16.1.03 (Statgraphics Technologies, The Plains, VA, USA), and the experiments were conducted randomly to minimize bias. Subsequently, multiple response variables were optimized using the desirability function, in which r and Υ were maximized.

Milli-Q water was heated in a thermostatic bath to reach the solution temperature of each polysaccharide to prepare the FS. Gly was added, and the mixture was stirred magnetically for 15 min. Then, the polysaccharide was slowly added to the solution while stirring magnetically at the solution temperature of each hydrocolloid for approximately 1 h until complete dissolution. Once the polysaccharide was completely dissolved, the solution was cooled to 20 °C and dispersed using an Ultraturrax (IKA T18, Staufen, Germany) at 10,000 rpm for 5 min. The resulting mixture was degassed with an ultrasound bath for 15 min to remove air bubbles. Finally, the FS was stored refrigerated for further analysis.

#### 2.2.3. Edible Compound Coatings (ECC)

For the preparation of ECC, the optimal concentrations of each polysaccharide obtained from the factorial design were used. Subsequently, a Simplex Lattice mixture design was conducted to evaluate the effects of two components (AS and FS) in eight randomly chosen runs to minimize the impact of hidden variables. The design was carried out in a single block, as shown in Table 2. The evaluated response variables were r, Υ, and MPS. The software STATGRAPHICS Centurion XVI, v.16.1.03 (Statgraphics Technologies, The Plains, VA, USA) was employed for the mixture design. Subsequently, multiple optimizations of the response variables were performed using the desirability function, in which r and Υ were maximized and minimized, respectively.

Each formulation was magnetically stirred at room temperature (20 °C) and 1400 rpm for 1 h, followed by homogenization for 15 s using an Ultraturrax (IKA T18, Staufen, Germany) at 3000 rpm.

#### 2.2.4. Edible Compounds Coatings Solid (ECCS)

The optimal mixtures of ECC (TLS/FS-HPMC and TLS/FS-KCG) were used to prepare ECCS and determine their mechanical properties. To prepare the ECCS, 30 mL of each mixture was deposited on square polystyrene plates measuring 10 × 10 cm and dried at 25 °C for 48 h in a forced convection oven (LabTech Model LDO-150F, Incheon, Republic of Korea). After drying, the ECCS was conditioned with a saturated solution of sodium bromide (58% relative humidity) at 25 °C for 48 h inside a sealed chamber. FS-HPMC and FS-KCG were used as controls, respectively. The mechanical properties of the ECCS were evaluated through tensile and puncture tests using a texture analyzer (Zwick/Roell BDO-FBO.5T5, Einsingen, Germany).

#### 2.2.5. Surface Properties

The ECC prepared with the methodology described in Section 2.2.3 of this manuscript was used to evaluate the surface properties of the coatings, such as density, surface tension, surface roughness, contact angle, and color.

##### Density

The density of the ECCS was determined using a 10 mL pycnometer and expressed in the International System of Units (SI). The density value was calculated using Equation (1).

(1) $$\rho =\frac{m}{V}$$

where:

*ρ*: Density of the sample at room temperature of 20 °C, [kg/m^3^].
*m*: Mass of the ECC sample deposited in the pycnometer, [kg].
*V*: Volume of the pycnometer, [m^3^].

##### Surface Tension

The pendant drop method [32] was used to determine the surface tension of the ECC. A high-speed camera (Plunix Inc., San José, CA, USA) coupled with a programmable syringe pump was used to regulate the ECCs flow and form a constant droplet. The system utilized HotShot 1.3 software (NAC Image Technology INC, Tokyo, Japan) to capture 20 images per sample, which were later analyzed using MATLAB R2013A 8.1.0, 604 software (The Math-Works Inc., Natick, MA, USA). The surface tension of each sample was obtained using the Laplace equation.

(2) $$\frac{dsen\theta }{dx}=\frac{2}{b}-\frac{g∆\rho }{\Upsilon }*\;z-\frac{sen\theta }{x}$$

where:

z,x: Cartesian coordinates at any point on the pendant drop.
b: Radius of curvature at the vertex.
g: Acceleration due to gravity.
∆ρ: Density difference between the sample and air at room temperature.
θ: Angle between the axis of the droplet and the normal to the droplet interface.
Υ: Surface tension between the sample and air.

##### Surface Roughness

To measure the roughness of the hydrophobic surface (HPS) and polydimethylsiloxane (PDMS), we employed the white light interferometry (WLI) technique using a Profilm3D^®^ optical profilometer equipped with a Nikon 10x CF IC Epi Plan objective and a Nano series active vibration isolation system from Accurion^®^. Data processing were performed using ProfilmOnline^®^ software (Filmetrics, San Diego, CA, USA). The root mean square (RMS) roughness value and peak-to-valley parameters were used to characterize the surface determined from images, with a field of view of 1500 × 1500 [µm^2^] according to the ISO 25178-1:2016 standard [33].

##### Contact Angles

The contact angles of the ECC were measured on an HPS and a PDMS surface at room temperature (20 °C) using a high-resolution optical system (Edmund Optics, Barrington, NJ, USA). Drops of 20 µL of the sample were deposited on each surface using a micropipette. ImageJ software determined the contact angle between the tangent plane to the drop surface and the tangent plane to the surface [34]. The spreading coefficient (S), the adhesion energy (*W_a_*), and the cohesion energy (*W_c_*) were calculated using Equations (3)–(5), respectively.

(3) $$S=W_{a}-W_{c}$$

(4) $$W_{a}=\Upsilon (1+cos\theta )$$

(5) $$W_{c}=2\Upsilon$$

where:

Υ: Surface tension between the ECC and air at a temperature of 20 °C.
θ: Angle between the axis of the droplet and the normal to the droplet interface.

##### Color

The color analysis of the EC was performed using a HunterLab colorimeter (HunterLab, Reston, VA, USA), which employs a D65 illuminant to simulate daylight. The color was expressed in terms of the L*, a*, and b* parameters according to the CIELab scale. The color difference (∆E*) between the ECC and the control FS was calculated using Equation (6).

(6) $${∆E}^{*}=\sqrt{{{(∆L}^{*})}^{2}+{{(∆a}^{*})}^{2}{{+(∆b}^{*})}^{2}}$$

where:

${∆E}^{*}$: Total color difference between the ECC Sample and the base color standard FS.
${(∆L}^{*})$: Difference in luminosity between the ECC sample and the base color standard FS.
${(∆a}^{*})$: Shift in the green-red color between the ECC sample and the base color standard FS.
${(∆b}^{*})$: Shift in the blue-yellow color between the ECC sample and the base color standard FS.

##### Rheology

To determine the rheological properties of the samples, a rheometer (Discovery Hybrid Rheometer HR2, TA Instruments, New Castle, DE, USA) with a cone and plate configuration (1.008°; 60 mm diameter; and a 27 μm gap between the geometry and the plate) was used. The lower plate was equipped with a Peltier temperature control system. 1 mL of the sample was deposited on the lower plate, and the upper plate was lowered to a position 27 μm above it. The system was then allowed to stabilize for 5 min at the initial working temperature. An oscillatory amplitude test was conducted to determine the linear viscoelasticity range of each sample. Once this region was determined, the oscillatory tests were carried out with a temperature sweep from 5 °C to 80 °C (ascending) and from 80 °C to 5 °C (descending) at a heating/cooling rate of 2 °C/min, deformation in the linear viscoelasticity range, and a 1 Hz angular frequency [35].

#### 2.2.6. Mechanical Properties

The ECCS was prepared with the methodology described in Section 2.2.4 of this manuscript was used to evaluate the mechanical properties, such as film thickness and tensile and puncture tests. The ECCS has a laminated appearance with light yellow and orange tones due to the presence of tannins. The TLS control has a gel appearance. Three repetitions were made for each film.

##### Thickness

The thickness of the ECCS was measured using a digital micrometer (Mitutoyo, QUICKmini, Kawasaki, Japan).

##### Tensile Testing

For the tensile test, strips measuring 2.5 cm in width and 8 cm in length were cut, and the thickness was measured at both ends and in the center of the strip. A texture analyzer (Zwick/Roell BDO-FBO.5T5, Einsingen, Germany) equipped with tensile grips was used to determine the mechanical properties of ECCS using the standard method ASTM D882-18 [36,37]. The initial distance between the grips (*L*_0_) was set to 60 mm with a crosshead speed of 10 mm/s. The instrument records the force (N), elongation or deformation (Δ*L*, mm), and test time (s).

The following mechanical properties were evaluated:

Elastic modulus (*E*), [Pa].

Maximum tensile force (*F_max_*), [N].

Tensile strength (*σ_max_*), [Pa].

Break force (*F_break_*), [N].

Tensile stress at break (*σ_break_*), [Pa].

Elongation at break (*EAB*) percentage.

The cross-sectional area of each ECCS was calculated using Equation (7).

(7) $$A_{T}=width\times thickness=A_{T}[m^{2}]$$

The tensile stress (*σ*) was determined from the force data using Equation (8).

(8) $$\sigma =\frac{F}{A_{T}}$$

where:

*F*: Tensile load force (N).

The unit strain (*ε*) was calculated using the Equation (9).

(9) $$\varepsilon =\frac{\Delta L}{L_{0}}$$

where:

Δ*L*: Deformation, [m].
*L*_0_: Initial distance, [m].

A stress-strain curve was constructed using the data of tensile stress (*σ*) and strain (*ε*), where the slope of the linear region of the curve (elastic zone) corresponds to the modulus of elasticity (E) or Young’s modulus [Pa]. Based on the force data provided by the equipment, the maximum force (*F_max_*) was determined, which corresponds to the force before fracture, the maximum length (*L_max_*), and the breaking force (*F_break_*) at which the ECCS broke. The tensile strength (*σ_max_*), the tensile stress at fracture (*σ_break_*), and the percentage of elongation (*EAB*, Elongation at break) were determined using Equations (10)–(12), respectively.

(10) $$\sigma_{max}=\frac{F_{max}}{A_{T}}$$

(11) $$\sigma_{break}=\frac{F_{break}}{A_{T}}$$

(12) $$EAB=\frac{L_{max}-L_{0}}{L_{max}}\times 100[\%]$$

##### Puncture Testing

Puncture tests were conducted to evaluate the coatings’ puncture resistance (RP) and *E_p_*. Circular samples with a 3 [cm] diameter were cut, and the thickness was measured at five points. The ECCS specimens were positioned in a 2.5 [cm] diameter cell, and force was applied until fracture occurred using a stainless-steel plunger-type probe (5 mm diameter) at a speed of 60 mm/min [38]. The maximum applied force (*F_max_*) was recorded during the test. Puncture resistance (RP) and *E_p_* were calculated using Equations (13) and (14), respectively.

(13) $$RP=\frac{F_{max}}{A_{T}}$$

(14) $$E_{p}=\frac{\sqrt{\left(r^{2}+d^{2}\right)}-r}{r}\cdot 100$$

where:

*F_max_*: Maximum applied force [N].
*A_T_*: Cross-sectional area of ECCS located in the cell (*A_T_* = 2re), [m^2^].
*r*: Radius of ECCS, [m].
*e*: Thickness of ECCS, [m].
*d*: Displacement of the probe from the contact point with ECCS to the point of fracture, [m].

##### Effective Diffusivity (Def)

The effective diffusion coefficient (Def) [m^2^⁄s] was determined using the analytical equation derived from Fick’s second law to determine the mechanism of tannin release from the ECCS subsequently.

(15) MtM∞=42d∗Def·tπ12

where:

*Mt*/*M∞*: Mass Fraction of tannins (catechin or epicatechin) that has been released at time *t*, [h].
*d*: Average thickness of ECCS, [m].
*t*: Time, [s].

##### Study of Tannin Release from ECCS

To investigate the release of tannins encapsulated in liposomes and their mixtures with polysaccharides, the concentration of catechin and epicatechin from ECCS was measured at time intervals (0; 5; 30 min; 1; 4; 8; 12; 24, 72, and 144 h). A piece of ECCS measuring 2 × 2 [cm^2^] was weighed and placed in a Falcon tube containing 9 [mL] of simulant for fatty foods (50:50 ethanol/water (%*v*/*v*)), following Regulation 10/2011 of the European Union. Subsequently, the tubes were kept at a constant temperature of 20 °C and shaken at 400 [rpm] in an orbital shaking incubator (JJBiotek JSSI-100C, Bangalore, India). The release was quantified using ultra-high-performance liquid chromatography (UHPLC), employing a C18 column and a UV detector at 280 nm. The obtained data were then fitted to the kinetic models of Higuchi (Equation (16)), Peppas & Sahlin (Equation (17)), Ritger & Peppas (Equation (18)), Korsmeyer & Peppas (Equation (19)), and Lindner & Lippold (Equation (20)) using the statistical software Curve Expert Professional [39,40].

(16) $$Mt/M\infty =K_{1}*t^{1/2}$$

(17) $$Mt/M\infty =K_{1}*t^{n}+K_{2}*t^{2n}$$

(18) $$Mt/M\infty =K_{1}*t^{1/2}+K_{2}*t$$

(19) $$Mt/M\infty =k*t^{n}$$

(20) $$\frac{Mt}{M\infty }=K_{1}*t^{n}+b$$

where:

*Mt*/*M∞*: Mass Fraction of tannin (catechin or epicatechin) that has been released at time *t*, [h].
*n*: Exponent indicating the release mechanism of tannins.
*K*_1_: Represents the contribution of the Fickian mechanism.
*K*_2_: Represents the contribution to the relaxation mechanism of the polymeric chains.
*b*: Represents the tannin immediately released upon contact with the simulant medium.

#### 2.2.7. Statistical Analysis

All experiments were performed in triplicate, and the experimental data obtained were expressed as the mean ± standard deviation. The software automatically verifies the normal distribution of the samples through normal probability plots [41]. Differences between three or more groups were evaluated using one-way or two-way ANOVA tests, followed by Tukey’s post hoc tests with a confidence level of 95% (*p* < 0.05) to determine statistical significance. The experimental designs and mathematical model fittings were verified using the coefficient of determination (R^2^), standard deviation, and residual analysis. All statistical analyses were conducted using the software STATGRAPHICS Centurion XVI, v.16.1.03 (Statgraphics Technologies, The Plains, VA, USA).

## 3. Results

### 3.1. Characterization of the Antioxidant Suspension (AS)

The results of the analyzed antioxidant suspension, corresponding to the TLS shown in Table 3, provide relevant information about their physicochemical characteristics.

The physicochemical characterization of the TLS revealed crucial insights. The suspension exhibited an MPS of 259.7 ± 5.10 nm, indicative of nanometer-sized particles, while a low PDI of 0.26 ± 0.03 suggested uniform particle distribution. The negative ξ of −41.8 ± 1.50 mV highlighted the surface charge responsible for colloidal stability. Notably, the catechin and epicatechin content were 91 ± 0.02% and 79 ± 0.01%, respectively, contributing to the TPC of 77.96 ± 2.42 mg AG/100g. The antioxidant capacity, evaluated by ABTS and ORAC assays, was reflected in values of 12.09 ± 0.034 μmoles TE/100g and 1187.14 ± 1.24 μmoles TE/100g, respectively. These findings underscore the suspension’s potential to deliver powerful antioxidant properties for various applications.

### 3.2. Optimization of the Filmogenic Suspension (FS)

**Figure 1:**
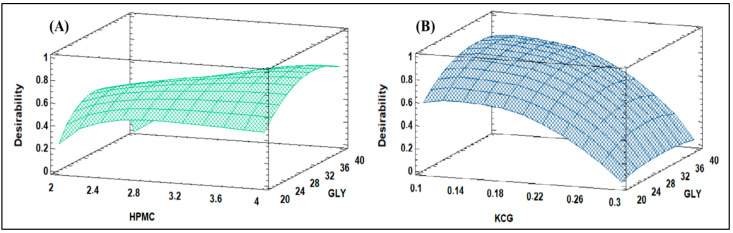
Estimated response surface for filmogenic suspensions of HPMC (**A**) and KCG (**B**).

Figure 1 illustrates the estimated response surface for the film-forming suspension of two different biopolymers, HPMC and KCG. The response surface plots visually represent how the concentration of the biopolymers and other components in the filmogenic suspension influences specific properties or outcomes.

In Figure 1A, the response surface represents the film-forming suspension containing HPMC. The plot’s axes depict the concentrations of HPMC and GLY within the suspension. The contour lines, or surface contours, illustrate how changes in the concentrations of these components affect the response variable being measured. This response variable could be a specific property related to the film formation, such as thickness, mechanical strength, or surface tension.

Similarly, Figure 1B shows the response surface for the film-forming suspension containing KCG. The same principle applies: the concentrations of KCG and GLY are represented on the axes, and the contour lines or surface contours demonstrate the relationship between these concentrations and the response variable.

The plots aid in identifying optimal conditions for achieving desired coating characteristics and can guide the formulation and optimization of these materials for specific applications.

### 3.3. Design of Simplex Lattice Mixture

Section 3.3 of this study involves the design of a simplex lattice mixture, where the polysaccharides HPMC and KCG were analyzed for variability using mixture designs. The results demonstrated that first- and second-order models were successfully fitted to individual responses. The p-values associated with these models were found to be less than 0.05, indicating that the models were appropriately fitted to the data.

**Figure 2:**
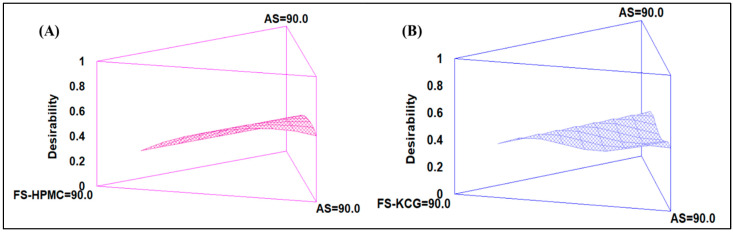
Optimization of multiple responses applied to mixtures of TLS/FS-HPMC (**A**) and TLS/FS-KCG (**B**).

In Figure 2A, the mixture under investigation is TLS/FS-HPMC, and the mixture proportions of these components are evaluated. The figure likely showcases a graphical representation of the optimization process, illustrating how varying proportions of the components lead to changes in multiple responses. These responses could include density, surface tension, mechanical properties, or other relevant characteristics of the resulting edible coatings.

Similarly, Figure 2B focuses on the mixture TLS/FS-KCG, presenting the same optimization process applied to this combination. Again, the figure is expected to demonstrate how altering the mixture proportions of TLS and FS-KCG influences multiple responses associated with the properties of the coatings.

These graphical representations aid in identifying optimal conditions for the mixture compositions that result in desirable coating properties.

### 3.4. Surface Roughness

**Figure 3:**
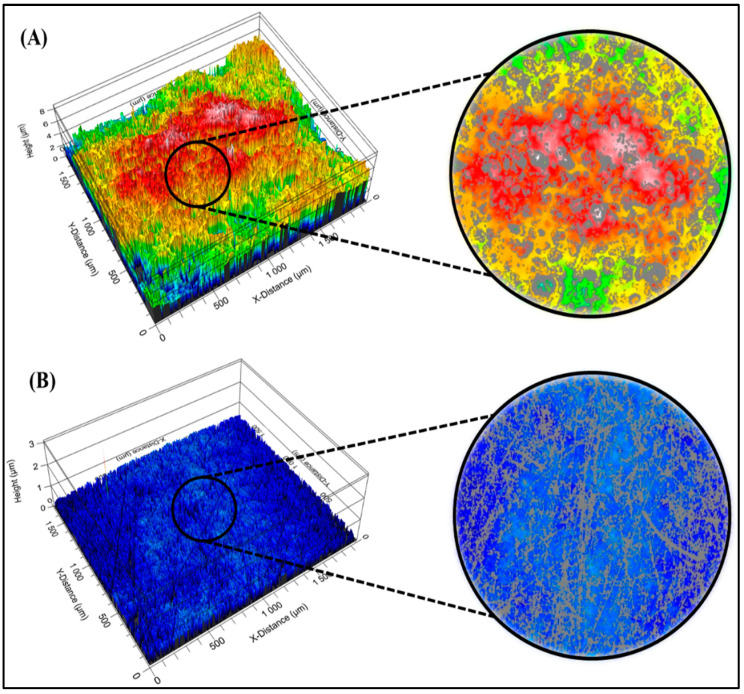
Surface roughness images of HPS (**A**) and PDMS (**B**) obtained by WLI.

Figure 3 shows the surface roughness images obtained using white light interferometry (WLI). The surfaces being examined are the HPS and PDMS surfaces. This analysis aims to assess the roughness characteristics of these surfaces using two specific roughness parameters: the root mean square roughness (RMS) and the peak-to-valley difference.

Figure 3A shows the surface roughness image of HPS. This image visually represents the surface’s texture and irregularities, which can impact its properties such as adhesion, friction, and wear. The RMS value quantifies the average height variations across the surface, indicating its overall roughness level. Additionally, the peak-to-valley difference signifies the vertical distance between the highest peak and the lowest valley on the surface.

Figure 3B illustrates the surface roughness image of PDMS. Similar to the previous image, this one visually shows the surface texture and irregularities. The RMS value and peak-to-valley difference of the PDMS surface are also evaluated to characterize its roughness features.

White light interferometry is a non-contact technique used to measure surface topography with high precision. The images and roughness parameters obtained from this analysis provide valuable insights into the surfaces’ physical characteristics, which can affect their performance and interactions in various applications.

### 3.5. Surface Properties of ECC

Table 4 shows the results for the surface properties of different ECCs. Five samples were analyzed: TLS, HPMC, KCG, and coatings formulated with nanoliposomes and HPMC (TLS/FS-HPMC) and nanoliposomes and KCG (TLS/FS-KCG). The properties studied include density, surface tension, dispersion coefficients on different surfaces, and color differences of the mixtures concerning each control.

### 3.6. Rheological Properties of ECC

**Figure 4:**
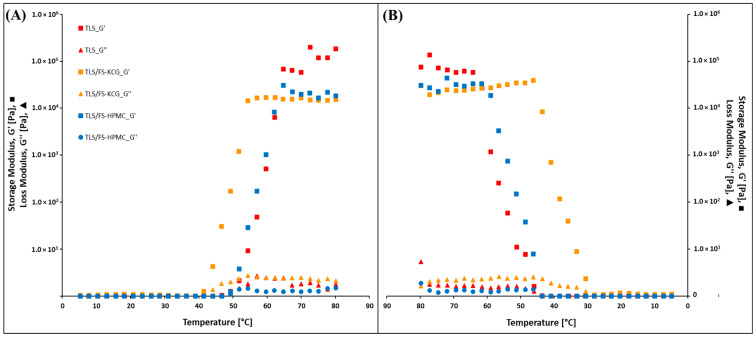
Upward temperature sweep from 5–80 °C (**A**) and downward temperature sweep from 80–5 °C (**B**) for the studied ECC.

Figure 4 shows the changes in storage modulus (G′) and loss modulus (G″) concerning temperature variations for the different coating samples, specifically TLS, TLS/FS-HPMC, and TLS/FS-KCG. The temperature range for this investigation spans from 5 °C to 80 °C in an upward temperature sweep from 5–80 °C (A) and a downward temperature sweep from 80–5 °C (B).

G′ and G″ are viscoelastic properties that describe a material’s ability to store and dissipate energy under deformation. G′ indicates the material’s elasticity or stiffness, while G″ signifies its viscosity or energy dissipation capacity.

These results provide a comprehensive understanding of how the studied edible coatings respond to temperature variations and the associated changes in their viscoelastic properties. Such insights are valuable for food preservation and packaging applications, as temperature changes can significantly impact the mechanical characteristics and performance of these coatings.

### 3.7. Mechanical Properties of ECCS

Table 5 shows the results of the mechanical properties of different ECCS. Five samples were analyzed: TLS, HPMC, KCG, and coatings elaborated with nanoliposomes and HPMC (TLS/FS-HPMC) and nanoliposomes and KCG (TLS/FS-KCG). The properties studied include film thickness, Young’s modulus, stress at break, percentage elongation at break (EAB), resistance to puncture (RP), and percentage elongation (Ep).

### 3.8. Tannins Release Study from ECCS

**Figure 5:**
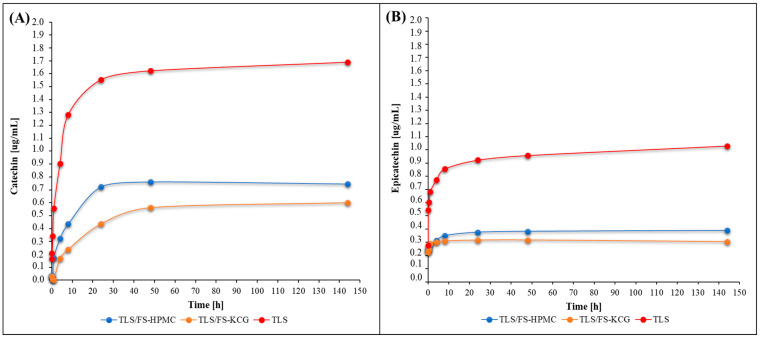
Catechin release from ECCS and TLS (**A**) and epicatechin release from ECCS and TLS (**B**).

Figure 5 shows the concentrations of catechin and epicatechin released from edible coating samples over time. Figure 5A displays the release of catechin from ECCS and the reference sample TLS. On the other hand, Figure 5B depicts the release of epicatechin from both the ECCS and TLS.

Catechin and epicatechin are active compounds with antioxidant properties found in coatings. Monitoring their release over time provides insights into the kinetics of compound release and how these compounds interact with the coating materials. The release profiles can indicate the coating’s potential effectiveness in delivering these active compounds to food products, thus contributing to their antioxidant and functional properties.

Understanding the release kinetics of these compounds is crucial for optimizing the coatings’ formulation and application. This knowledge can be valuable for developing coatings that provide targeted releases of bioactive compounds to enhance food quality and shelf life.

## 4. Discussion

### 4.1. Characterization of the Antioxidant Suspension (AS)

The MPS of the TLS was 259.7 ± 5.10 [nm]; this is relevant considering the structure of condensed tannins, which can aggregate and form colloidal dispersions with hydrodynamic diameters ranging from nanometers to microns [42]. During sonication, the cavitation forces generated by the ultrasonic probe, along with the microflow forces and turbulence produced during ultrasound cycles, promote particle size reduction [43]. This size reduction is beneficial in biological applications, as smaller particles have greater absorption capacity and biodisponibility in the human body [44]. The PDI analysis showed a value of 0.26 ± 0.03, which is favorable as low polydispersity indicates a more uniform distribution of particles in the sample [45]. Regarding the electric charge of the particles, a negative ξ of −41.8 ± 1.50 [mV] was observed; this negative charge can be attributed to the presence of functional groups on the surface of the particles, providing colloidal stability to the sample and preventing aggregation due to electrostatic repulsion [46]. A similar study encapsulating myrtle extract in nanoliposomes presented an MPS value of 293.17 ± 3.15 nm, a PDI of 0.37 ± 0.01, and a zeta potential of −31.80 ± 0.10 mV [47]. In addition to the physical characteristics of the sample, the results also reveal a high encapsulation efficiency of 91% for catechin monomer and 79% for epicatechin monomer in the analyzed sample, indicating that nanoliposomes have an excellent capacity to protect and effectively deliver these active compounds. The antioxidant capacity of TLS was evaluated using ABTS and ORAC. The results showed that the antioxidant capacity measured by ORAC was significantly higher (*p* < 0.05) than ABTSs. This discrepancy can be attributed to differences in the measurement mechanisms of both methods, as they can detect different types of antioxidants or antioxidant reactions with different sensitivities and detection ranges. Therefore, it is likely that nanoliposomes containing grape seed tannins exhibit higher antioxidant activity according to the ORAC method, implying a greater capacity to neutralize free radicals and protect against oxidative stress [48].

### 4.2. Optimization of the Filmogenic Suspension (FS)

The results obtained in Figure 1A show that the optimal concentration of the HPMC-based film-forming suspension (FS-HPMC) was 4%, with 33% Gly and a desirability value of 0.79. In addition, Figure 1B shows that the optimal value of the KCG-based film-forming suspension (FS-KCG) was 0.1%, with 35% Gly and a desirability value of 0.89. These results suggest that adding Gly to both coating suspensions is essential to achieve a higher desirability value, indicating better final product quality. Additionally, optimizing HPMC and KCG concentrations will improve the film-forming capacity of the solution and its texture, which are essential in applications in the food industry.

### 4.3. Design of Simplex Lattice Mixture

The optimal mixture of TLS/FS-HPMC was 65% TLS and 35% FS-HPMC, while the optimal mixture of TLS/FS-KCG was 75% TLS and 25% FS-KCG; for this, the response variables density and surface tension were maximized. Higher density can contribute to the stability of the colloidal system and improve coating coverage [49], while higher surface tension can promote the formation of tiny droplets with better dispersion [50]. In summary, the results indicate that the linear fit for HPMC and the quadratic model for KCG are suitable for modeling the variability of these solutions, and the determined optimal mixtures can improve the quality of the final product in specific applications.

### 4.4. Surface Roughness

On the HPS surface, an RMS value of 1.126 [µm] and a peak-to-valley difference of 6.933 [µm] were obtained. The high peak-to-valley difference suggests significant variations in the height of the peaks and valleys, indicating high roughness compared to the PDMS surface. In addition, much lower values were recorded on the PDMS surface for both RMS (0.055 µm) and peak-to-valley difference (2.989 µm), indicating that the PDMS surface is smoother and exhibits less variability than the HPS surface. Sobhani [51] studied the mechanical and surface properties of HT-PDMS elastomer and reported an RMS value between 0.049 and 0.054 [µm], indicating minimal roughness due to the particles present on the surface. Dopilka [52] reported an RMS value of 0.030 [µm] for a thin PDMS silicone film. These results suggest that the HPS surface is rougher and more irregular than the PDMS surface. These characteristics, shown in Figure 3, could be relevant and related to the surfaces’ adhesion, cohesion, and wetting properties [53].

### 4.5. Surface Properties of ECC

The density values ranged from 1010 to 1050 [kg/m^3^], and statistically significant differences (*p* < 0.05) were observed among all samples. These differences may be attributed to the varying compositions of each sample. The lowest value was obtained by HPMC, which is related to the quantity of hydroxyl and methyl groups present in the molecule. This variation can affect density due to changes in the average molecular mass [54]. When comparing the film-forming suspensions, the structure of kappa carrageenan might lead to more significant swelling than HPMC, potentially contributing to the higher density of KCG [55]. Moreover, the higher density value among the composite coatings (ECC) was achieved by TLS/FS-HPMC, suggesting greater strength or thickness of the coating. Conversely, the lower density of TLS/FS-KCG indicates lower resistance and higher elasticity [56].

Furthermore, the surface tension values of the edible coatings composed of TLS/FS-HPMC and TLS/FS-KCG showed significant differences (*p* < 0.05) compared to the control sample of TLS nanoliposomes. In the case of the HPMC mixture, a decrease in surface tension was observed, suggesting improved wetting and spreading ability on the surface, which could be beneficial for achieving uniform coverage [57]. Anyway, in the case of the KCG mixtures, an increase in surface tension was observed, which could reduce the formation of cracks and defects in the coating and promote better cohesion, decreasing the likelihood of detachment [58,59].

The scattering coefficients of the samples in HPS (S_1_) ranged from −2.9 to −17.6, whereas in PDMS (S_2_), lower values were obtained, ranging from −17.6 to −40.9. Negative scattering coefficient values suggest cohesive properties at the interfaces of the samples [60]. The higher magnitude (more negative) values of S_2_ than S_1_ could indicate a lower tendency to spread at the interface, implying that they have a reduced inclination to extend over the surface. Thus, the PDMS surface, being silicone and highly flexible, allows for less sample spreading [61].

The color difference measured through the ∆E2000 parameter for the samples TLS, TLS/FS-HPMC, and TLS/FS-KCG, along with their respective controls TS, HPMC, and KCG, exhibited statistically significant differences (*p* < 0.05). These differences in the ECCs can be attributed to the presence of tannins, which are colored compounds [62]. The TLS sample displayed the lowest ∆E2000 value, indicating a lesser chromatic disparity with its reference [63], while TLS/FS-HPMC exhibited the highest value. These results are obtained because TLS/FS-HPMC contains 35% FS-HPMC, which alters its color since the filmogenic suspensions are transparent.

### 4.6. Rheological Properties

In the case of the TLS/FS-HPMC coating, G′ increased as the temperature rose, suggesting an increase in material rigidity at higher temperatures. This phenomenon can be attributed to stronger bonds or interactions between the material’s molecules [64]. Similarly, G″ also exhibited an increase with temperature, indicating a greater capacity for energy dissipation by specific molecular structures of the polymer, which could be associated with viscous behavior or the formation of a thermal gel [65]. For TLS/FS-KCG, a similar G pattern was observed with increasing temperature. However, regarding G″, a general decrease was noted as temperature increased, implying that the coating material has lower stiffness and a higher elastic recovery capacity than TLS/FS-HPMC [66]. In addition, TLS exhibited a lower ability to store elastic energy, indicating lower structural rigidity [67].

It is essential to highlight that all three materials responded similarly to temperature changes, indicating that they do not exhibit thixotropy, meaning any changes in their viscoelastic properties over time [68].

### 4.7. Mechanical Properties of ECCS

The samples did not exhibit significant differences (*p* < 0.05) in the film thickness values. The TLS sample exhibited a Young’s modulus value of 6.00 × 10^7^ [Pa], indicating relatively low stiffness compared to HPMC and the TLS/FS-HPMC film, which had a Young’s modulus value of 2.00 × 10^8^ [Pa]. These results suggest that the presence of TLS in the ECCS does not alter the polymer’s rigidity [69]. The TLS/FS-HPMC sample maintained good tensile strength while exhibiting higher deformation capacity before rupture [70].

Anyway, the KCG and TLS/FS-KCG films showed rupture strengths of 3.00 × 106 [Pa] and 4.00 × 106 [Pa], respectively. Elongation before rupture was 94.71 ± 0.296 [%] for KCG and 96.49 ± 0.839 [%] for TLS/FS-KCG. These results suggest that KCG coatings have similar mechanical properties to each other, with high deformation capacity before rupture. Concerning puncture resistance, the outcomes indicated that HPMC exhibited the highest resistance at 18.18 ± 0.294 [Pa], followed by TLS/FS-HPMC, KCG, TLS/FS-KCG, and TLS in decreasing order. These differences were statistically significant (*p* < 0.05) and may be attributed to the nature of the employed polysaccharides and the percentage of incorporated TLS in the ECCS. Regarding the elongation percentage, TLS showed the highest value (*p* < 0.05), indicating a high deformation capacity before fracture, which may be attributed to the ductility of the lipids present in the nanoliposomes, implying their ability to stretch significantly before breaking [71].

### 4.8. Tannins Release Study from ECCS

The TLS sample showed rapid and sustained release of catechins, followed by stabilization in the concentration of epicatechin. Additionally, the diffusivity of TLS exhibited an initial increase followed by a constant decrease over time.

TLS/FS-HPMC and TLS/FS-KCG mixtures exhibited different release patterns. The TLS/FS-HPMC coating showed progressive and continuous release for both tannin monomers, with a gradual increase in diffusivity over time, reaching a maximum value of 4.13 × 10^−14^ [m^2^/s] for the first hour of contact with the fatty simulant medium, followed by a decrease and stabilization at six days. The TLS/FS-KCG coating showed slower initial release, followed by a significant increase in catechin concentration and stabilization of epicatechin. It exhibited an initial increase in diffusivity with a maximum value of 1.59 × 10^−14^ [m^2^/s] for 5 min in contact with the simulant medium and then decreased to stabilize at 3.97 × 10^−15^ [m^2^/s] at six days.

These differences in release profiles could be explained by the differences in the chemical structure of each polysaccharide and coating component [72] or the formation of complexes or interactions with the active compound and the coating materials acting as retaining agents [73]. The mathematical fitting of five previously described kinetic models in this report was applied to analyze these results further. The Peppas & Sahlin model best fits the catechin and epicatechin release curves in all samples, with high coefficients of determination (R^2^) in the range of 0.977 to 0.985. This model is based on the power law and provides a more complex description of release mechanisms, incorporating relaxation and erosion phenomena inherent to viscoelastic polymeric materials [74].

## 5. Conclusions

ECCs’ mechanical and surface properties formulated with TLS, HPMC, and KCG were analyzed. The results revealed that the components’ composition influenced the coatings’ surface and mechanical properties. Including HPMC or KCG affected the density, surface tension, and scattering coefficients, which impact the coating’s adhesion, cohesion, and wettability. In addition, differences in mechanical properties were observed between the ECCS, where the TLS/FS-HPMC showed higher stiffness and springback than the TLS/FS-KCG. Regarding the release of tannins, a more rapid release of catechin was noted than that of epicatechin, indicating the influence of chemical and structural properties on the release of the compounds. This study provides solid foundations for developing ECC with applications in foods susceptible to oxidation. The implications include the shelf life and quality improvement of these products. Future perspectives include optimization of formulations, exploration of specific applications, investigation of new bioactive compounds, and interaction studies with food. In summary, this study contributes to the field of biopolymer-based functional materials by providing insight into how the components interact and affect the properties of ECCs, with potential for applications in the food and packaging industries.

## Figures and Tables

**Table 1:** Multilevel factorial design *.

Factors	Low	High	Levels	Units	Response	Units
HPMC	2	4	3	[%*w*/*v*]	Density	[kg/m^3^]
KCG	0.1	0.3	3	[%*w*/*v*]	Surface tension	[mN/m]
Gly	20	40	3	[%*w*/*w*] relative to polysaccharide		

* Software STATGRAPHICS Centurion XVI, v.16.1.03 (Statgraphics Technologies, The Plains, VA, USA).

**Table 2:** Simplex Lattice Design for mixtures with polysaccharides *.

Block	TLS	FS-HPMC	TLS	FS-KCG	Response	Units
1	70	30	90	10	Density	[kg/m^3^]
1	10	90	10	90	Surface tension	[mN/m]
1	50	50	30	70	Mean particle size	[nm]
1	90	10	70	30		
1	30	70	50	50		
1	90	10	50	50		
1	10	90	90	10		
1	90	10	50	50		

* Software STATGRAPHICS Centurion XVI, v.16.1.03 (Statgraphics Technologies, The Plains, VA, USA).

**Table 3:** Physicochemical characterization of TLS *.

MPS [nm]	PDI	ξ [mV]	Catechin [%]	Epicatechin [%]	TPC [mg AG/100 g]	ABTS [μmoles TE/100 g]	ORAC [μmoles TE/100 g]
259.7 ± 5.10	0.26 ± 0.03	−41.8 ± 1.50	91 ± 0.02	79 ± 0.01	77.96 ± 2.42	12.09±0.034	1187.14 ± 1.24

* The results correspond to mean ± standard deviation.

**Table 4:** Surface properties of ECC *.

Sample	Density [kg/m^3^]	Surface Tension (Υ) [mN/m]	Scattering Coefficient (S_1_), HPS [mN/m]	Scattering Coefficient (S_2_), PDMS [mN/m]	∆E_2000_
TLS	1041 ± 0.577 ^d^	55.49 ± 1.247 ^d^	−2.935 ± 0.087 ^e^	−17.642 ± 0.482 ^d^	9.98 ± 0.007 ^a^
HPMC	1010 ± 0.577 ^a^	47.88 ± 0.745 ^b^	−15.281 ± 0.060 ^b^	−18.033 ± 0.048 ^d^	-
TLS/FS-HPMC	1050 ± 0.577 ^e^	42.42 ± 1.040 ^a^	−8.601 ± 0.268 ^d^	−22.788 ± 0.573 ^c^	20.46 ± 0.263 ^c^
KCG	1021 ± 1.155 ^b^	53.74 ± 1.453 ^c^	−9.820 ± 0.370 ^c^	−30.484 ± 0.945 ^b^	-
TLS/FS-KCG	1030 ± 1.528 ^e^	66.09 ± 0.241 ^d^	−17.626 ± 0.184 ^a^	−40.958 ± 0.379 ^a^	14.74 ± 0.036 ^b^

* The results correspond to mean ± standard deviation. Different letters in the same column indicate significant differences (*p* < 0.05).

**Table 5:** Mechanical properties of ECCS *.

Sample	Thickness [mm]	Young’s Modulus [Pa]	$\sigma_{break}[Pa]$	EAB [%]	RP [Pa]	Ep [%]
TLS	0.16 ± 0.012 ^a^	6.00 × 10^7 a^	3.00 × 10^6 a,b^	93.73 ± 1.533 ^b^	2.06 ± 0.211 ^a^	147.86 ± 4.295 ^b^
HPMC	0.16 ± 0.022 ^a^	2.00 × 10^8 d^	1.00 × 10^7 c^	91.06 ± 0.354 ^a^	18.18 ± 0.294 ^d^	14.69 ± 1.441 ^a^
TLS/FS-HPMC	0.16 ± 0.016 ^a^	2.00 × 10^8 d^	9.00 × 10^6 c^	94.71 ± 0.296 ^b,c^	13.74 ± 0.944 ^c^	16.25 ± 2.350 ^a^
KCG	0.17 ± 0.011 ^a^	8.00 × 10^7 b^	3.00 × 10^6 a^	94.74 ± 2.019 ^b,c^	7.40 ± 1.199 ^b^	17.36 ± 4.678 ^a^
TLS/FS-KCG	0.17 ± 0.036 ^a^	1.00 × 10^8 c^	4.00 × 10^6 b^	96.49 ± 0.839 ^c^	7.02 ± 0.838 ^b^	21.85 ± 5.108 ^a^

* The results correspond to the mean ± standard deviation. Different letters in the same column indicate significant differences (*p* < 0.05).

## Data Availability

Data are available upon request.
